# Misconceptions about paraoxonase-1

**DOI:** 10.1016/j.bjorl.2021.08.009

**Published:** 2021-10-26

**Authors:** Michael Mackness, Eser Y. Sozmen

**Affiliations:** a Avenida Princip D’Espanya, Tarragona, Spain; b Ege University Faculty of Medicine, Department of Medical Biochemistry, Bornova, Turkey

Dear Editor,

We have read the manuscript “The relationship between thiol-disulfide balance and idiopathic sudden sensorineural hearing loss” in press in BJORL^1^ with interest and recognized that the discussion on paraoxonase activity (Paragraph 4) contained a number of factually inaccurate statements. Firstly, the authors state that paraoxonase and arylesterase are different enzymes. Human serum Paraoxonase (PON1) is classified as an aryldialkylphosphatase (EC 3.1.8.1), has the capability to hydrolyse various substrates including lactones, thiolactones, organophosphorus triester pesticides and nerve gases, arylesters, oestrogen-esters, cyclic carbamates and glucuronide drugs^2^ and has a physiological role in many diseases such as inflammation, organophosphate intoxication, drug metabolism and cardiovascular disease.^3^ A series of elegant experiments conducted in Prof Bert La Du’s laboratory in Michigan over 20 years ago showed that in humans paraoxon and phenylacetate were substrates of the same enzyme i.e. PON1. These studies were subsequently confirmed by laboratories around the world at the biochemical, molecular biological and molecular genetic levels.^2^, ^3^, ^4^ Paraoxonase and arylesterase are therefore 2 activities of PON1.

Secondly, the authors probably mistakenly stated that paraoxonase is a “lipid metabolism product” and “PON1 is an organophosphate”. However, it is clear from the literature that paraoxonase is an enzyme which hydrolyses oxidised lipids.^4^ Therefore PON1 is not a lipid oxidation product. Nor is it an organophosphate, again PON1 is an enzyme, organophosphates are its substrates.^2^, ^3^, ^4^

Finally, the authors also state “Elevated PON levels are related to atherosclerosis”. The reference given to justify this statement (citation 19 in Ref.^1^) is one of ours and we can assure you the opposite is true. Low PON is related to atherosclerosis.

These inaccuracies may seem minor to those not in the field; however, they are important and need correcting before they have time to spread.

## Conflicts of interest

The authors declare no conflicts of interest.
